# High-Frequency and High-Power Performance of *n*-Type GaN Epilayers with Low Electron Density Grown on Native Substrate

**DOI:** 10.3390/ma15062066

**Published:** 2022-03-11

**Authors:** Roman M. Balagula, Liudvikas Subačius, Justinas Jorudas, Vytautas Janonis, Pawel Prystawko, Mikolaj Grabowski, Irmantas Kašalynas

**Affiliations:** 1 Terahertz Photonics Laboratory, Center for Physical Sciences and Technology, Saulėtekio al. 3, LT-10257 Vilnius, Lithuania; liudvikas.subacius@ftmc.lt (L.S.); justinas.jorudas@ftmc.lt (J.J.); vytautas.janonis@ftmc.lt (V.J.); 2 Laboratory of Semiconductor Characterization, Institute of High Pressure Physics PAS (UNIPRESS), ul. Sokołowska 29/37, 01-142 Warsaw, Poland; pprysta@unipress.waw.pl (P.P.); mgrabowski@unipress.waw.pl (M.G.)

**Keywords:** gallium nitride with low electron density, shallow silicon impurity, low-field electron mobility, electrical performance, strong pulsed electric field

## Abstract

The *n*-type GaN epilayers with low electron density were developed on a native substrate using the metalorganic vapour phase epitaxy method and investigated under pulsed electric fields until material breakdown and optically in the spectrum range from 0.1 THz to 60 THz at two temperatures of 77 K and 300 K. The epilayers demonstrated the low-field electron mobility and density values reaching up to 1021 cm^2^/V·s and 1.06 × 10^16^ cm^−3^ (at 300 K) and 2652 cm^2^/V·s and 0.21 × 10^16^ cm^−3^ (at 77 K), respectively. Maximum injected electric power value till the damage of the GaN epilayer was found to be up to 1.8 GW/cm^3^ and 5.1 GW/cm^3^ at 77 K and 300 K, respectively. The results indicate new practical possibilities of the GaN material controlled by an external electric field.

## 1. Introduction

The high quality of gallium nitride (GaN) epilayers is crucial for the fabrication of high-power and high-frequency semiconductor devices [1,2,3,4,5]. Existing growth technologies are already sufficient for the development of high-electron mobility transistor (HEMT) heterostructures [6,7] and various vertical GaN-on-GaN devices such as MOSFET transistors [8], Schottky diodes [4,9,10], and p-n diodes [5,11]. Free electron density plays important role in such epilayers, which is why the use of silicon (Si) is preferable for *n*-type doping in low density cases to counteract unintentional doping by oxygen (O) and carbon (C) impurities in a controllable manner. As a result, the density of free electrons in the GaN layer can be reduced down to a level of ~10^16^ cm^−3^. As of now, the best parameters for such applications on a native substrate were obtained by pulsed sputtering deposition [1,12], molecular beam epitaxy [2,3,13], and metalorganic vapour phase epitaxy (MOVPE) [14] with the smallest values for electron density of 2 × 10^16^ cm^−3^, 9.2 × 10^15^ cm^−3^, and 9.9 × 10^15^ cm^−3^, respectively. Carrier mobility is another important parameter for the discussed structures, with the highest low-field mobility values at low temperatures reported to be 3470 cm^2^/V·s, 2637 cm^2^/V·s, and 6660 cm^2^/V·s, respectively, while at room temperature it remained above 1000 cm^2^/V·s in all cases. The reason for such high electron mobility was the low density of threading dislocations (TDD) in the used substrates, the values of which were reported to be (2–4) × 10^6^ cm^−3^ [1,12,14], 4 × 10^7^ cm^−3^ [13], (0.5–5) × 10^9^ cm^−3^ [3], and 2 × 10^6^–2 × 10^10^ cm^−3^ [2]. Ammonothermal GaN crystals possess TDD as low as 5 × 10^4^ cm^−3^ [15], which could allow achieving even higher carrier mobilities in the epilayers grown on these substrates. However, if TDD is below 2 × 10^6^ cm^−2^ and doping of the GaN epilayer is above 2 × 10^16^ cm^−3^, the numerical modelling shows that scattering on ionized impurities (at cryogenic temperatures) and on polar optical phonons (at room temperatures) dominates among other mechanisms that could limit the electron mobility [2,12]. Shallow impurities in GaN such as Si and O warrant type and quantity of dopants in the material, which define optical and electric properties of epilayers, and the characterization of which can be performed using various non-destructive methods [16,17,18]. The processes of impurity incorporation and resulting electronic and electric properties of the material have been efficiently investigated in A_3_B_5_ semiconductors by applying density-functional theory calculations [19,20,21,22].

This work is devoted to the investigation of optical and electrical performances of *n*-GaN epilayers grown on an ammonothermal GaN substrate using the MOVPE method at room and liquid nitrogen temperatures. Our results demonstrate high low-field mobility and low density of electrons in studied epilayers, which can be used for the development of GaN-based devices for high-power and high-frequency applications.

## 2. Experimental Details

A 10 μm-thick GaN epilayer was grown by a MOVPE method on a semi-insulating (SI) GaN substrate, with the total thickness *d* measured with a micrometer being 380 ± 10 μm. Lateral ohmic contacts and isolation pads were formed by using standard UV photolithography and reactive ion etching (RIE) processing technologies.

**Figure 1:**
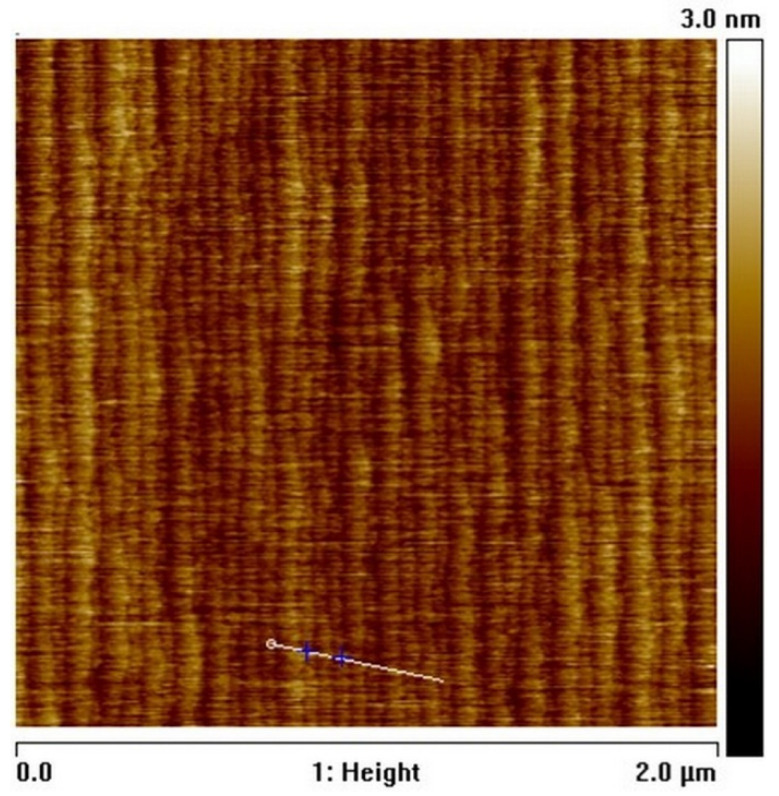
AFM image of as-grown surface of the GaN epilayer.

First, after loading into the Close Coupled Showerhead (CCS^TM^) Aixtron 3 × 2-inch flip top (FT) reactor (Aixtron, Herzogenrath, Germany), the GaN substrate was annealed in order to remove oxygen and silicon accumulated at the surface while avoiding the crystal atomic steps desorption. Optimized annealing conditions resulted in a very fast decay of contaminants toward the growth direction. Typically, oxygen and silicon drop below 8 × 10^15^ cm^−3^ within the first 40 nm, as measured by secondary ion mass spectrometry (SIMS) analysis. Next, the main layer of GaN was grown at a temperature of 1060 °C, reactor pressure of 500 torr, and a high V/III ratio. These conditions provide low carbon incorporation from the gas phase, promoting fast recombination of methyl radicals originating from the trimethylgallium (TMGa) source material to methane. The 10 μm-thick epilayer was nominally doped with silicon to 1.29 × 10^16^ cm^−3^ using SiH_4_ gas, while the actual carbon concentration was kept below 6 × 10^15^ cm^−3^ as evidenced by SIMS analysis. A highly n-type doped 200 nm-thick GaN subcontact layer, doped with silicon to 3 × 10^18^ cm^−3^, was deposited on top. During the test device fabrication, this subcontact *n*+ layer was selectively removed from all areas except the metalized patterns with the dry etching (Cl-based RIE, Oxford Plasmalab-100 ICP tool, Oxford Instruments, Abingdon, UK) technique. A group of the 11 μm-deep mesa structures was also fabricated using dry etching; therefore, the resulting structures were isolated within the semi-insulating ammonothermal GaN material. The Ti/Al/Ni/Au (30/90/20/150 nm) metal stacks evaporated using Denton Vacuum Explorer 14 *e*-beam (Denton Vacuum, Moorestown, NJ, USA) and annealed at 850 °C in Jipelec JetFirst RTP furnace (ECM Technologies, Grenoble, France) were used as Ohmic contacts. All dry-etched GaN surfaces were wet treated in tetramethylammonium hydroxide (TMAH) solution. An image of the as-grown surface obtained using atomic force microscopy (AFM) is shown in Figure 1. The AFM image of the GaN epilayer revealed usual monolayer atomic steps, the height of which were approximately 0.27 nm. By definition, RMS roughness can be assumed as half of the step (<0.14 nm). To be more specific, the surface roughness was measured at different sites of the *n*-GaN epilayer, resulting in the average value of *R*_q_ = 0.168 ± 0.017 nm.

**Figure 2:**
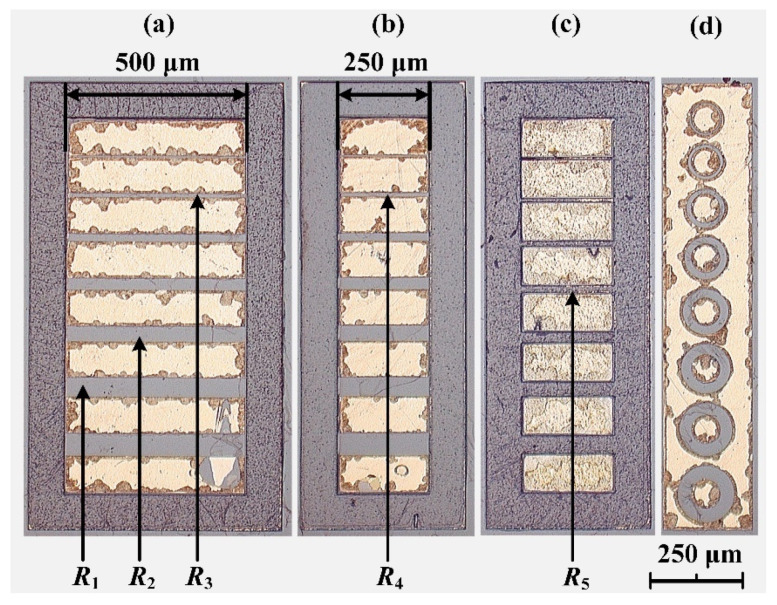
Photograph of ohmic contact pads fabricated on the sample obtained using optical microscope: (**a**) wide TLM structures (W-TLM) with contact width of 500 μm, (**b**) narrow TLM structures (N-TLM) with contact width of 250 μm, (**c**) I-TLM structures with contact width of 250 μm and removed GaN epitaxial layer between the contact pads, and (**d**) CTLM with diameter of central contact pad of 80 μm. Labels *R*_1_, *R*_2_, and *R*_3_ denote resistors on the W-TLM structures with contact separation of 55 μm, 45 μm, and 12.5 μm, respectively. Label *R*_4_ denotes the resistor on the N-TLM structure with contact separation of 12.5 μm. Label *R*_5_ denotes the I-TLM structure with contact separation of 20 μm.

The sample was characterized using electrical and optical methods. Hall measurements were performed in Van der Pauw geometry using the Ecopia HMS-3000 Hall Effect Measurement System (Ecopia, Anyang, South Korea) employing fixed magnetic field *B* = 0.55 T in the regime of low electric fields and currents ranging from 1 µA to 1 mA at two temperatures of 300 K and 77 K. The values of carrier density and mobility obtained at 4 different current values in the range specified above were averaged, finding the mean and standard deviation values. Temperature dependence of resistance was measured in the range of 4–300 K using the current limit of 1 mA set by the Keithley 2400 (Keithley Instruments, Solon, OH, USA) source-measure unit (SMU). The performance of the ohmic contacts was investigated using the transmission line method (TLM) and circular TLM (CTLM) techniques [23]. Resistor geometries for the former and latter techniques are shown in Figure 2a,b and Figure 2d, respectively. Contact separations in rectangular TLM structures were 6, 12.5, 25, 35, 45, 55, and 65 μm, while in CTLM, contact separations ranged from 5 μm to 40 μm with a step of 5 μm. Fabricated resistors with two contact pads of various shapes, widths, and spacings between them were investigated on the probe station Cascade Microtech MPS 150 (Cascade Microtech, Beaverton, OR, USA), connecting the selected resistor to the SMU. Rectangular-shaped isolation TLM (I-TLM) structures were also designed with the same separation distances between the pads as in TLM structures, but without the *n*-type GaN epilayer, which was etched out by RIE (see Figure 2c). I-TLM structures served for the measurement of lateral breakdown voltage of the GaN bulk substrate, connecting the selected structure with two pads to the high-voltage SMU.

**Figure 3:**
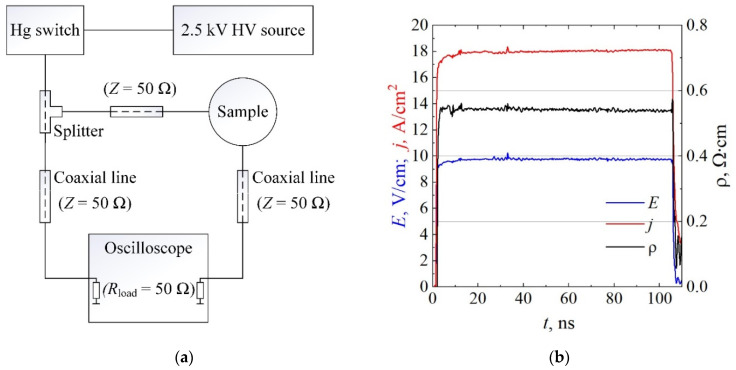
(**a**) Electrical scheme of I/V characteristic measurements in the regime of nanosecond duration pulses of electric field. (**b**) Example of shape of electric field, current density, and resistivity pulsed traces obtained at low electric field.

Pulsed current–voltage (I/V) characteristics were measured in a scheme matched to 50 Ω impedance using a Hg relay that delivered voltage pulses with duration, repetition rate, and rise time of about 110 ns, 100 Hz, and 0.5 ns, respectively. An electrical scheme of the measurements is shown in Figure 3a. Short voltage pulses were applied to avoid the effects of heat accumulation in the crystal lattice. The sample was placed in a holder designed for measurements in a 50 Ω impedance circuit. A high-frequency digital oscilloscope LeCroy Wave-Surfer 7200 (Teledyne LeCroy, Chestnut Ridge, NY, USA) with a bandwidth of 2 GHz was used to measure traces of voltage and current pulses. The samples were immersed in liquid nitrogen for low-temperature measurements at *T* = 77 K. Typical pulsed traces of the current density, electric field, and resistivity of the sample are shown in Figure 3b. The values of current density and electric field were obtained, taking into account the cross-section area (product of width and depth) and the length of the particular resistor under test, respectively.

Optical characterization was performed at *T* = 300 K. In the infrared (IR) range, the transmission and reflection spectra were obtained by a Fourier-transform infrared (FTIR) spectrometer Thermo Scientific Nicolet 8700 (Thermo Fisher Scientific, Waltham, MA, USA) operating in a rapid-scan mode with a KBr beam-splitter and a DLaTGS pyroelectric photodetector. A terahertz time-domain spectroscopy (THz TDS) system Teravil T-SPEC 800 (Teravil, Vilnius, Lithuania) purged with dry nitrogen was used for measurements of the transmission spectra in the THz range. In both cases, the sample was attached to a free-standing metal aperture with diameter of 2 mm. Transmission was obtained in the zone of the GaN epilayer that was not affected during the fabrication of test devices with removed 200 nm-thick subcontact layers. Modelling of THz transmission was conducted using the transfer matrix method with dielectric function derived from the Drude conductivity model, a detailed description of which can be found elsewhere [24,25].

## 3. Results and Discussion

**Figure 4:**
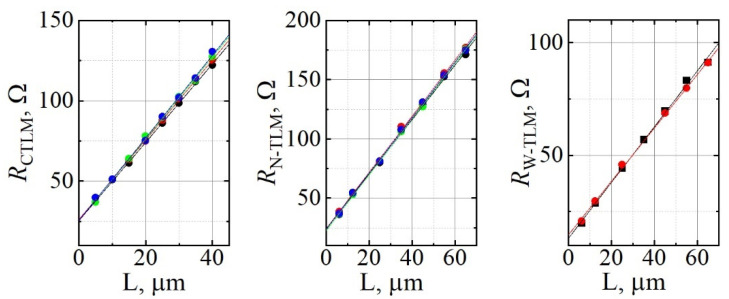
CTLM, N-TLM, and W-TLM graphs for resistance dependence on contact separation distance. Symbols represent measurement data and solid lines indicate linear fit.

Characterization of ohmic contacts to the epilayer was carried out at room temperature using TLM and CTLM structures positioned throughout the wafer. The results of electrical measurements are shown in Figure 4.

The averages and standard deviations of extracted parameters are presented in Table 1. Small values of standard deviation for all parameters indicate good uniformity of contact and epilayer material properties across the wafer. Low contact resistance allows considering the results obtained by contact methods to be reliable. It is worth noting that good performance of the ohmic contacts to GaN epilayers with low free electron density is necessary to avoid uncertainties in the interpretation of impurity breakdown characteristics [16].

**Figure 5:**
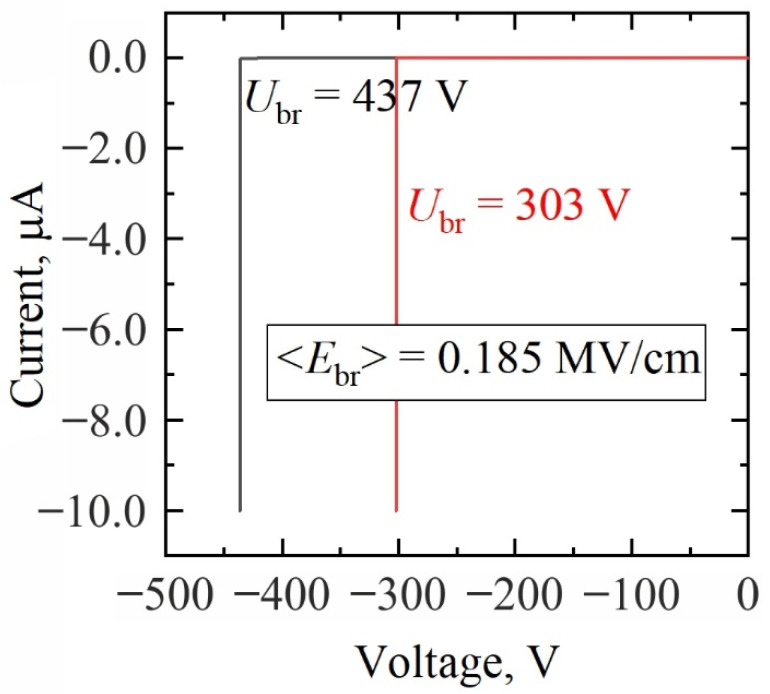
Lateral breakdown voltage measurements of the GaN bulk after device processing. I-TLM structures with 20 μm contact separation (labelled as *R*_5_ in Figure 2c) at different locations on the wafer were used.

Measurements of lateral electrical breakdown were performed on I-TLM structures with electrical resistance values being in the range of 200 GΩ. Testing on a few isolation structures of various lengths revealed the average breakdown field to be 0.19 ± 0.05 MV/cm. Selected results are shown in Figure 5. The found value of the lateral breakdown field of the GaN bulk after device processing was comparable to the results previously reported for Schottky barrier diodes longer than 30 μm [26] and GaN epilayers [27]. Although the published data on this parameter vary strongly with measurement conditions, device geometry, and structure, the measured value was sufficient for the characterization of GaN epilayer performance under strong electric fields.

**Figure 6:**
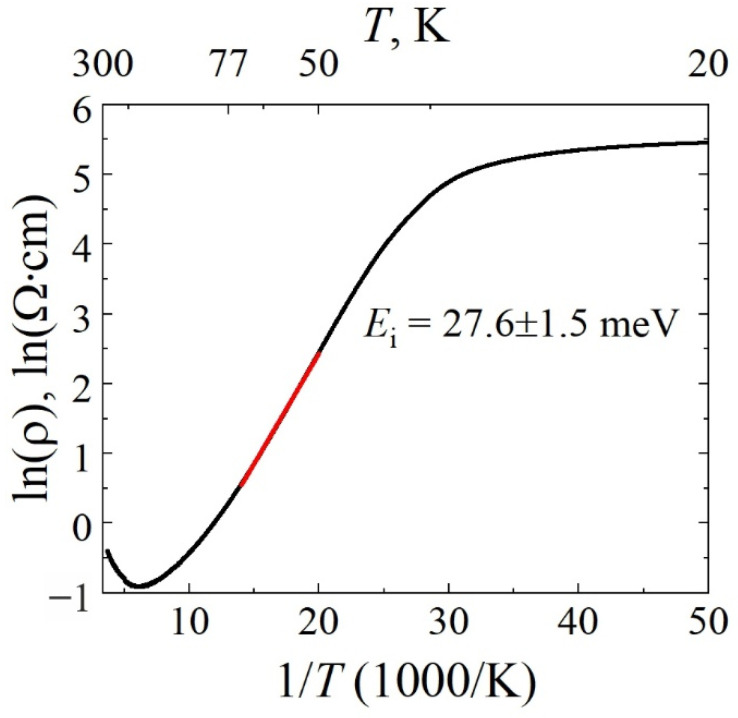
Arrhenius plot of temperature dependence of resistivity for a resistor *R*_1_. Value of activation energy, *E*_i_, obtained by a linear fit in the temperature range of 50–70 K is indicated. It is worth noting that the resistor at *T* = 80 K, 158 K, and 300 K demonstrated resistivity values of 1.16 Ω·cm, 0.4 Ω·cm, and 0.71 Ω·cm, respectively.

Temperature dependence of resistivity was obtained for different contact pairs with separation varying from 12 μm to 55 μm. A characteristic dependence for a resistor *R*_1_ plotted in Arrhenius coordinates is shown in Figure 6. Assuming that change in low-field electron mobility is small [14], and taking into account equation $\rho =\rho_{0}\times \exp\left(–E_{d}/k_{B}T\right)$, impurity activation energy was obtained by a linear fit of experimental data in the temperature range of 50–70 K with the value of *E*_i_ = 27.6 ± 1.5 meV. Such a value corresponds to the activation energy of the silicon impurities in GaN material [18,28]. Note that similar energies were observed by THz electroluminescence spectroscopy of Si impurities in epilayers with and without HEMT structures [16,17].

**Figure 7:**
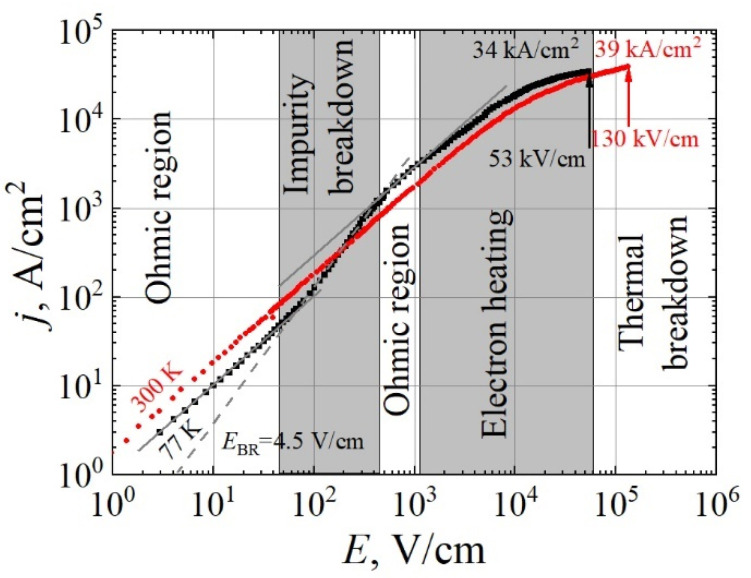
Pulsed current density versus electric field characteristics of *n*-type GaN epilayer at *T* = 77 K (Resistor *R*_1_ with the length and width of 55 μm and 500 μm, respectively; black squares) and 300 K (Resistor *R*_2_ with the length and width of 45 μm and 500 μm, respectively; red circles). At 77 K, distinct features attributed to impurity ionization and carrier heating were observed in the region of ~38–450 V/cm and fields higher than ~1200 V/cm, respectively. Breakdown of the resistor occurred at the electric field of about 53 kV/cm. At 300 K, the I/V curve is linear up to electric fields of ~1.5 kV/cm, followed by electron heating and eventual breakdown at the electric field of 130 kV/cm. Maximum injected electric power till the damage of GaN epilayer was found to be up to 1.8 × 10^9^ W/cm^3^ and 5.1 × 10^9^ W/cm^3^ at 77 K and 300 K, respectively.

**Figure 8:**
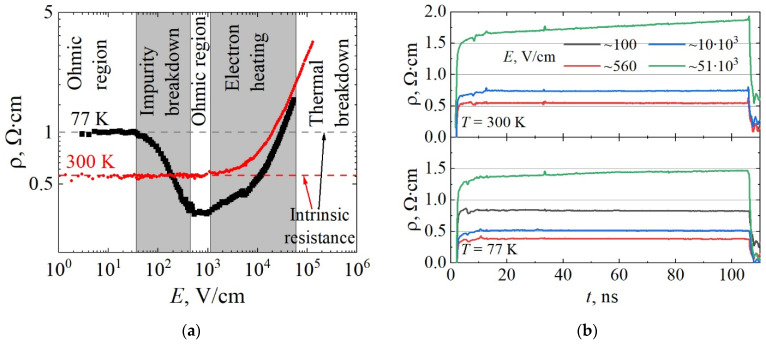
(**a**) Resistivity versus electric field characteristics obtained from the corresponding experimental data shown in Figure 7. (**b**) Resistivity kinetics during the electric pulse in various electric field regions: *E* ≈ 100 V/cm, black lines (impurity breakdown), *E* ≈ 560 V/cm, red lines (Ohmic), *E* ≈ 10 kV/cm, blue lines (electron heating), and *E* ≈ 51 kV/cm, green lines (electron and lattice heating with the rapid change in the latter during the entire pulse).

Pulsed I/V characteristics were measured for three resistors of the W-TLM structure shown in Figure 2a and one resistor of the N-TLM structure shown in Figure 2b. The lengths of the selected resistors were 12.5 μm, 45 μm, and 55 μm in W-TLM and 12.5 μm in N-TLM cases. The measured *j/E* characteristic of the 55 μm resistor *R*_1_ at 77 K is shown in Figure 7 with black squares. The resistivity electric field characteristic recalculated from *j/E* data is shown in Figure 8a. At 77 K, five distinct field regions were found and qualitatively attributed to the following processes: (i) linear increase in the current density demonstrating an intrinsic epilayer resistivity of 1.0 Ω·cm at very low electric fields; (ii) superlinear increase in current density owing to the breakdown of the impurities with activation energy and breakdown field, *E*_BR_, values of 27.6 meV and 4.5 V/cm, respectively, which is observed in the range from 38 V/cm to 450 V/cm; (iii) the second ohmic region in sample behaviour at electric fields higher than 450 V/cm, which demonstrates epilayer resistivity of 0.35 Ω·cm and constant carrier concentration due to fully ionized impurities, the density value of which can be measured at *T* = 300 K; (iv) sublinear increase in the current resulting mainly from a mobility decrease due to effect of the heating of electrons and phonons at fields above 1200 V/cm [29,30]; and (v) thermal breakdown of the epilayer material, which was found at the electric field of 53 kV/cm. At 300 K, the *j/E* curve measured for a 45 μm resistor *R*_2_ (red circles in Figure 7 and Figure 8a) is linear in the field range of 0–1.5 kV/cm, owing to the almost complete ionization of impurities with ionization energy close to *k*_B_*T* value at 300 K, with a further increase in the electric field sample demonstrating sublinear *j/E* characteristics due to electron heating and the eventual thermal breakdown at the field of 130 kV/cm.

The resistivity traces at different temperatures are shown in Figure 8b. Steady resistivity was measured during the 100 ns-long pulse of the electric field, while its value was below 15 kV/cm and 11 kV/cm at 77 K and 300 K, respectively. However, rapid change in the resistivity was observed at higher electric fields, indicating temperature accumulation in the nanosecond timescale.

Notably, at the temperature of 300 K, the critical electric field that damages the GaN epilayer was more than two times higher than that needed for material breakdown at 77 K. However, the critical current density values were similar in both cases, demonstrating the maximum potential of the GaN epilayers under research. The maximum pulse power density at which thermal breakdown occurs was found to be up to 1.8 GW/cm^3^ and 5.1 GW/cm^3^ at 77 K and 300 K, respectively. Note that, taking into account GaN epilayer thickness of 10 µm, the maximum current density per unit width of the material is estimated to be about 3.9 (3.4) A/mm at 300 K (77 K), which is considerably larger than the 2.4 A/mm values achieved in advanced devices based on the AlGaN/GaN HEMT structures at room temperature [31,32,33].

More precise data on the type of free carriers, their density and their mobility were obtained by Hall effect measurements at low electric fields. Electron-type conductivity with *n* = (1.06 ± 0.02) × 10^16^ cm^−3^, μ = 1021 ± 6 cm^2^/V·s and *n* = (0.21 ± 0.01) × 10^16^ cm^−3^, μ = 2652 ± 33 cm^2^/V·s was observed at *T* = 300 K and 77 K, respectively. At low electric fields, the electron density exhibits about a five-fold decrease with the reduction in temperature from 300 K to 77 K due to the freezeout of free carriers on shallow impurities. We note that the value of low field electron mobility is comparable to the highest values reported in the literature [1,2,3,12,13,14].

**Figure 9:**
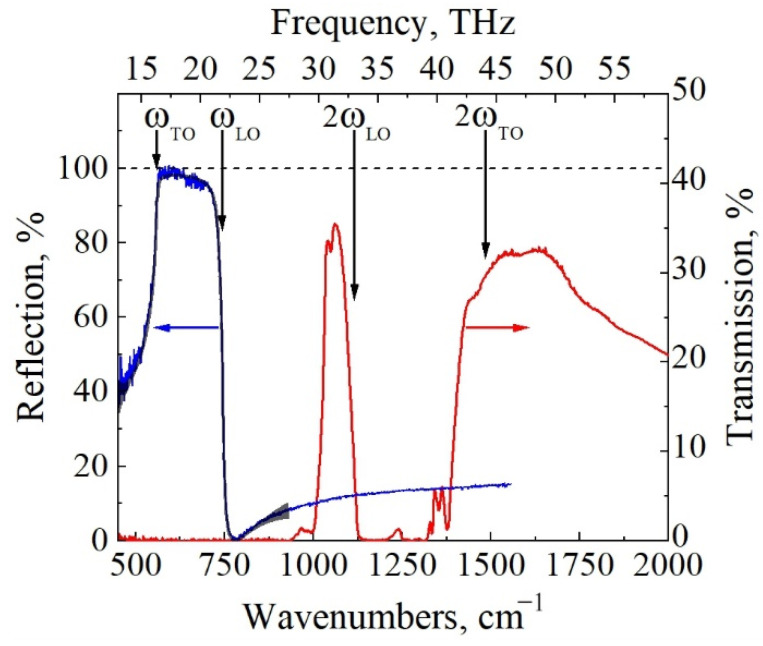
Experimentally obtained Transmission (red line) and Reflection (blue line) spectra of the *n*-type GaN epilayer. Modeled reflectivity spectrum (black line) was found by fitting experimental spectrum with bulk material parameters of ω_LO_ = 743 cm^−1^, ω_TO_ = 558 cm^−1^, γ_LO_ = 6.9 cm^−1^, γ_TO_ = 3.2 cm^−1^, *n* = 1.19 × 10^16^ cm^−3^, μ = 952 cm^2^/V·s, and *d* = 375 μm.

Optical characterization of GaN epilayer was also performed to corroborate the findings obtained using contact methods. Transmission of the sample was measured in the infrared (IR) spectral range for initial characterization. The obtained results are shown in Figure 9 with the red line. Several characteristic features can be identified as follows: (i) quite a small transmission in the spectrum range of ~25–30 THz, which was related to the light scattering due to the use of a one-side polished GaN substrate and (ii) a strong absorption region in the spectrum range of ~33–44 THz, attributed to the second harmonic of the Reststrahlen band of GaN, as indicated by vertical arrows at the frequencies of 2ω_TO_ and 2ω_LO_ [34]. The shape and slope of the shoulders of the two-phonon absorption band measured in transmission spectrum in this work are similar to those of GaN crystals reported in Ref. [35], demonstrating that the free-carrier density indeed should be below the level of ~4 × 10^17^ cm^−3^ (smallest doping value reported in [35]).

Experimentally measured reflection and numerically modelled reflectivity spectra of the GaN epilayer are shown in Figure 9 with blue and black lines, respectively. Modelling was performed using rigorous coupled-wave analysis as described in our recent publication [36]. The Reststrahlen band of GaN was well pronounced. Frequency of the longitudinal, ω_LO_, and transverse, ω_TO_, optical phonons with corresponding damping factors, γ_LO_, γ_TO_, free-carrier density, *n*, and mobility, *μ*, and the total thickness of the substrate with epilayer, *d*, were obtained from the fitting procedure, revealing the values to be ω_LO_ = 743 cm^−1^, ω_TO_ = 558 cm^−1^, γ_LO_ = 6.9 cm^−1^, γ_TO_ = 3.2 cm^−1^, *n* = 1.19 × 10^16^ cm^−3^, μ = 952 cm^2^/V·s, and *d* = 375 μm, respectively. Measured phonon frequencies and damping factors agree well with other findings reported in the literature [24,37]. It is worth noting that IR-active impurity mode with a characteristic frequency of about ω_IM_ = 736 cm^−1^ was included in the spectral analysis [38].

**Figure 10:**
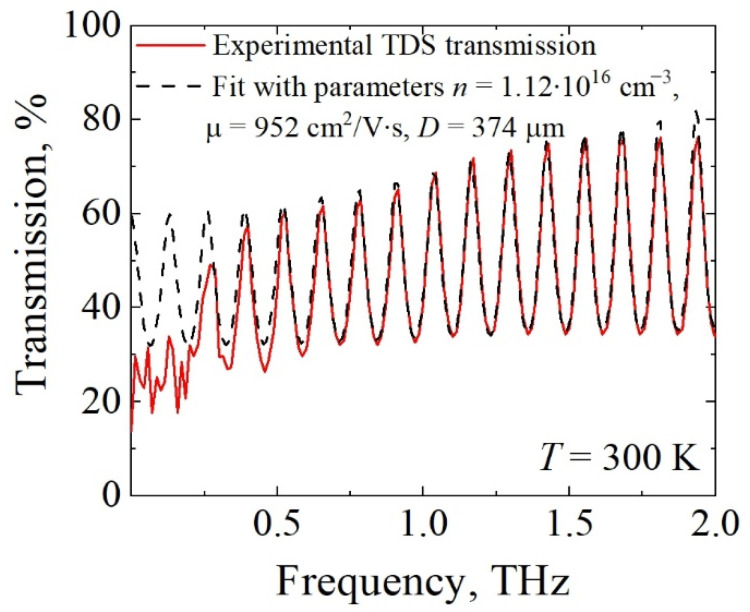
Experimental transmission spectrum measured by THz-TDS (solid red line) and calculated using transfer matrix method and high-frequency Drude conductivity of electrons (dashed black line). Results of fitting are also indicated.

Measured transmission spectrum of the GaN epilayer in the THz region is shown in Figure 10 with a red solid line. The contribution of free electrons was calculated using the high-frequency Drude conductivity model by adding the respective component to the dielectric function of the GaN epilayer [24]. The transfer matrix approach modified for conducting interfaces [39] was applied with electron density, mobility, and structure thickness as the fitting parameters. The results of fitting are shown in Figure 10 with a black dashed line. The modelling demonstrates good agreement with the experiment in the entire spectral range except for the frequencies below 0.3 THz, which is the result of the employment of a 2 mm aperture that blocks radiation with wavelengths longer than 1 mm. The aperture effect was not accounted for in numerical modelling. Fitting data and all findings at the low electric fields discussed above are summarized in Table 2. Values of parameters obtained using contact measurements and contactless optical methods are in a good agreement.

## 4. Conclusions

The performance of *n*-type GaN epilayers with low electron density grown on a bulk GaN has been investigated using the contact-based approach under pulsed electric fields till the breakdown of the material and the non-destructive optical characterization methods in the THz and IR frequencies, covering the spectrum region from 0.1 to 60 THz at two temperatures of 77 K and 300 K. The epilayers demonstrated the low-field electron mobility and density with values reaching up to 1021 cm^2^/V·s and 1.06 × 10^16^ cm^−3^ (at 300 K) and 2652 cm^2^/V·s and 0.21 × 10^16^ cm^−3^ (at 77 K), respectively. Layers were found suitable for the transfer of pulsed electric powers up to 5.1 × 10^9^ W/cm^3^ (at 300 K) and 1.8 × 10^9^ W/cm^3^ (at 77 K). The results demonstrate the potential of the MOVPE-grown GaN layers on native substrates in a wide temperature range and their suitability for the development of novel devices for high-power and high-frequency applications.

## Figures and Tables

**Table 1:** Contact resistance, transfer length, and sheet resistance of the GaN epilayer.

Contacts	*R*_c norm_, Ω·mm	*L*_T_, μm	*R*_sheet_, Ω/□
CTLM	3.24 ± 0.03	5.14 ± 0.12	630 ± 13
N-TLM	2.98 ± 0.08	5.09 ± 0.15	586 ± 7
W-TLM	3.49	5.75	606

**Table 2:** Summary of parameters of the *n*-type GaN epilayers measured at low electric field and by optical means.

**Van der Pauw**				
*T*, K	*n*, × 10^16^ cm^−3^	µ, cm^2^/V·s	ρ, Ω·cm	Thickness *d*, μm
300	1.06 ± 0.02	1021 ± 6	0.56	
77	0.21 ± 0.01	2652 ± 33	1.09	
**IR reflection**				
300	1.19	952	0.55	375
**THz TDS transmission**				
300	1.12	952	0.59	374

## Data Availability

Data available on request.
